# Is local platelet-rich plasma injection clinically superior to hyaluronic acid for treatment of knee osteoarthritis? A systematic review of randomized controlled trials

**DOI:** 10.1186/s13075-018-1621-0

**Published:** 2018-06-19

**Authors:** Yalong Di, Changxu Han, Liang Zhao, Yizhong Ren

**Affiliations:** 1 Department of Medical Imaging, Hebei General Hospital, Shijiazhuang, 050051 China; 2 Department of Sports Medicine, Second Affiliated Hospital of Inner Mongolia Medical University, Huhehaote, 010030 China

**Keywords:** Knee, Osteoarthritis, Platelet-rich plasma, Hyaluronic acid

## Abstract

**Background:**

In this study, we evaluated whether platelet-rich plasma (PRP) is superior to hyaluronic acid (HA) in the treatment of knee osteoarthritis.

**Methods:**

The Cochrane Central Register of Controlled Trials, PubMed, and Embase databases were searched for English-language, human in vivo studies on the treatment of symptomatic knee osteoarthritis with intra-articular PRP compared with HA. The following keywords were used for the search: “platelet-rich plasma,” “PRP,” “platelet-rich fibrin,” “PRF,” “platelet,” “plasma,” “arthritis,” “osteoarthritis,” “gonarthrosis,” and “degeneration.”

**Results:**

Seven articles reporting 908 patients and 908 knees were analyzed, including 44% men and 56% women with a mean age of 59.8 years. All studies met the minimal clinically important difference criteria and showed statistically significant improvements in clinical outcomes, including pain, physical function, and stiffness, with PRP treatment. All except two studies showed significant differences between PRP and HA regarding clinical outcomes of pain and function.

**Conclusions:**

PRP intra-articular injection of the knee may be an effective alternative treatment for knee OA, especially in patients with mild knee OA. Although some studies suggested that the effect of PRP was no better than HA, we found that it was no worse. A large, multicenter, randomized trial is needed to further assess the efficacy of PRP treatment for patients with knee OA.

**Trial registration:**

PROSPERO, CRD42016048394. Registered on October 2, 2016).

## Background

Osteoarthritis (OA) is a multifactorial chronic bone and joint disease characterized by articular cartilage degeneration that adversely impacts patient mobility and quality of life [1]. OA has been estimated to affect 27 million people in the United States [2]. In addition, the cartilage is avascular in this condition, and the cells have low mitotic activity. Healing potential is limited once the cartilage is injured, eventually leading to irreversible damage. These effects have a major impact on the functioning and independence of patients [2], especially the elderly. The prevalence of knee OA is 50% among patients aged above 65 years [3], and its main symptoms are knee pain, swelling, and limited mobility; furthermore, it is accompanied by a high prevalence of wide, late, and extensive functional disability.

The goal of treatment for knee OA is to relieve pain, improve function and quality of life, and reduce disability. Intra-articular injection of hyaluronic acid (HA) [4], corticosteroids, and platelet-rich plasma (PRP); oral nonsteroidal anti-inflammatory drugs; and physical therapy are important nonsurgical treatment options for knee OA. PRP is an autologous blood product produced by centrifugation of whole blood [5] that yields a concentration of platelets above the baseline value [6, 7].

PRP lacks proper standardization and definition. Differences between some of the key characteristics, including platelet concentration, anticoagulant and coagulation activation agent type, presence of inflammatory white blood cells, and activation level, can significantly affect the biological effect.

Local injection of autologous PRP in animal models has been shown to significantly improve the biomechanical behavior of cartilage and chondrocyte proliferation and to repair cartilage injury [8–10]. Although the relevant literature has moderate applicability and strength of evidence, the current guidelines of the American Association of Orthopedic Surgeons do not recommend or oppose the use of PRP in the treatment of knee OA. However, comparison studies conducted on the use of intra-articular injection of PRP compared with HA for mild or moderate knee OA showed a higher clinical outcome score with PRP than with the latter [11–14]. Therefore, the aim of this systematic review was to analyze randomized controlled trials (RCTs) of PRP and HA to determine whether PRP is superior to HA in the treatment of knee OA.

## Methods

### Research design

We conducted a systematic review in 2016 to investigate the effectiveness of PRP and HA for the treatment of knee OA.

### Study search

This systematic review was registered with PROSPERO on October 4, 2016 (registration ID CRD42016048394). The Preferred Reporting Items for Systematic Reviews guidelines were followed. The Cochrane Central Register of Controlled Trials (CENTRAL) (The Cochrane Library, 2016), PubMed, and Embase (January 2005 to August 2016) databases were searched for English-language, human in vivo studies on the treatment of symptomatic knee OA with intra-articular PRP in comparison with HA treatment. The following keywords were used for the search: “platelet-rich plasma,” “PRP,” “platelet-rich fibrin,” “PRF,” “platelet,” “plasma,” “arthritis,” “osteoarthritis,” “gonarthrosis,” and “degeneration.” In addition, presentations and abstracts from annual meetings of the American Academy of Orthopaedic Surgeons, the European League against Rheumatism, the American Academy of Physical Medicine and Rehabilitation, the American College of Rheumatology, and the Osteoarthritis Research Society International (OARSI) were manually searched. The search was performed independently by two reviewers. The search results were reviewed to determine which articles were ultimately included in the study according to inclusion criteria.

### Inclusion and exclusion criteria

Inclusion criteria for this study were as follows: (1) RCTs in which knee OA was identified; (2) studies that compared the use of autologous PRP with HA; (3) studies involving PRP and HA intra-articular injection; and (4) English-language, original, randomized comparative trials. The exclusion criteria were as follows: studies with unknown data and methodology and those conducted on patients with knee OA who had additional diseases, such as those with pain or swelling associated with knee joint disease, ligament or meniscus injury, arthritis, blood diseases, serious cardiovascular disease, or infection or those receiving immunosuppressive or anticoagulation therapy.

### Outcome measures

The main outcome of the efficacy and response to treatment for recovery used in this systematic review were the Western Ontario and McMaster Universities Osteoarthritis Index (WOMAC) [15], International Knee Documentation Committee (IKDC) [16], Knee Injury and Osteoarthritis Outcome Score (KOOS) [17], EuroQol visual analogue scale (EQ VAS) [18], and Tegner score [19].

### Data extraction

On the basis of inclusion and exclusion criteria of the study, two reviewers independently examined the titles and abstracts of studies. The selected studies were included in the systematic review. In case of a difference of opinion between the two reviewers, a third party acted as a referee, and the dispute was resolved by discussion. The following data were extracted from all eligible studies:

1. General study information: title, authors, publication year, and registration number
2. Study characteristics: study design, study setting, and inclusion/exclusion criteria
3. Details of the interventions: dose, frequency of administration, and duration of treatment
4. Primary and secondary outcome measures, including the results for the intervention and the comparison groups from baseline to follow-up, with the effect sizes [20]

The difference between the means, Cohen’s *d*, was calculated as follows: M1 − M2/s, where M is the mean value of either group and s is the standard deviation of either group. The other values calculated were the minimum clinically important difference [21] (with an effect size of 0.5) and *P* value.

Effect size (ES) is a name given to a family of indices that measure the magnitude of a treatment effect. Unlike significance tests, these indices are independent of sample size. ES measures are the common currency of meta-analyses that summarize the findings in a specific area of research.

### Quality assessment

Two independent reviewers assessed the quality of the included studies using the Cochrane Collaboration risk-of-bias tool as follows:

1. *Strong evidence*: Provided by at least two studies with a low risk of bias and by generally consistent findings in all studies (≥ 75% of the studies reporting consistent findings)
2. *Moderate evidence*: Provided by one study with a low risk of bias and/or at least two studies with a high risk of bias and by generally consistent findings in all studies (≥ 75% of the studies reporting consistent findings)
3. *Limited evidence*: Provided by only one study with a high risk of bias
4. *Conflicting evidence*: Inconsistent findings in multiple studies (≥ 75% of the studies reporting consistent findings)
5. *No evidence*: No studies found

## Results

### Search results

**Fig. 1:**
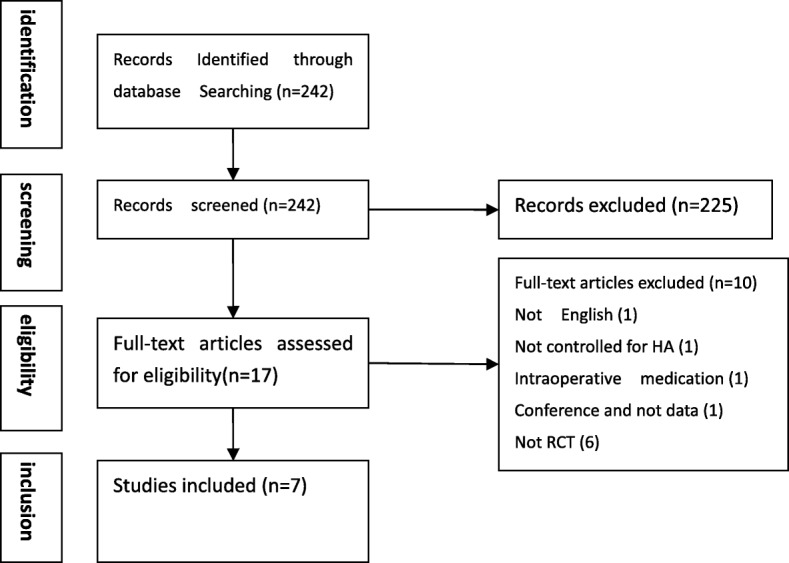
Search strategy results. *HA* Hyaluronic acid, *RCT* Randomized controlled trial

Of the 242 nonduplicate citations identified from the literature, 17 clinical trials were screened for eligibility (Fig. 1). Of these, 10 articles were excluded for the following reasons: introduction of PRP by arthroscopic surgery (not by injection) (one study), Chinese language (not English) (one study), assessment of PRP in comparison with placebo (not HA) (one study), conference proceeding that did not provide any data (one study), and non-RCTs (six studies).

### Description of studies

The characteristics of the included studies, excluded studies, and ongoing studies are provided in the online supplementary materials.

### Data analysis

All studies except those by Cerza et al. [22] and Filardo et al. [11] provided the registration numbers. In total, seven articles (908 patients, 908 knees) were analyzed (Table 1), and the study population included 44% men and 56% women with a mean age of 59.8 years. The number of injections and the interval and volume of PRP injection are shown in Table 1. The safety data, which summarize the adverse events for each study, are shown in Table 2.

**Table 1:** Demographics and methods of included clinical trials

	Cerza et al., 2012 [22]	Filardo et al., 2012 [11]	Sanchez et al., 2012 [24]	Vaquerizo et al., 2013 [25]	Filardo et al., 2015 [23]	Raeissadat et al., 2015 [13]	Montañez-Heredia, et al. 2016 [14]
Registered	Not recorded	Not recorded	Real Decreto 223/2004	Real Decreto 223/2004	ClinicalTrials.gov, NCT01670578	IRCT20140121134 42N5	ClinicalTrials.gov, NCT02448407
Subject enrollment date	September 2009–September 2010	Not recorded	January 2008–November 2009	Not recorded	2009–2013	Not recorded	January to March 2014
Country	Italy	Italy	Spain	Spain	Italy	Iran	Spain
Conflict of interest	None	None	None	Not mentioned	Not mentioned	None	None
No. of subjects (knees)	120 (120)	109 (109)	176 (176)	96 (96)	183 (183)	160 (160)	55 (55)
Sex: male, female	53, 67	68, 41	85, 91	38, 58	112, 71	23, 116	21, 32
Mean age, yr	66.4	56.5	59.8	63.6	55.34	58.79	63.9
BMI, kg/m^2^	Not recorded	26.5	28.0	30.9	25.7	27.68	29.7
Bilateral vs. unilateral knee injections	Unilateral	Unilateral	Unilateral	Unilateral	Unilateral	Unilateral	Unilateral
Ultrasound-guided injections or not	Not mentioned	Not mentioned	Not mentioned	Not mentioned	Not mentioned	Not mentioned	No
Study group 1	60 Patients	54 Patients	87 Patients	48 Patients	96 Patients	87 Patients	28 Patients
Study group 2	60 Patients	55 Patients	89 Patients	48 Patients	96 Patients	73 Patients	27 Patients
Baseline characteristic differences between groups	No differences	No differences	No differences	No differences	No differences except for age	No differences except for age, sex, WOMAC (pain, function)	No differences
Radiographic classification	Kellgren-Lawrence	Kellgren-Lawrence	Ahlbäck	Kellgren-Lawrence	Kellgren-Lawrence	Kellgren-Lawrence	Kellgren-Lawrence
	Grade I: 25	Average of grade	Grade I: 87	Grade II: 32	Average of grade	Grade I: 6	Grade I: 7
	Grade II: 22	2.2 for PRP group and 2.1 for HA group	Grade II: 64	Grade III: 47	2.0 for PRP group and 2.0 for HA group	Grade II: 91	Grade II: 19
	Grade III: 13		Grade III: 23	Grade IV: 17		Grade III: 75	Grade III: 27
						Grade IV: 28	
Length of follow-up	24 Weeks	12 Months	6 Months	48 Weeks	12 Months	52 Weeks	6 Months
Outcome scores used	WOMAC	IKDC, Tegner, KOOS, EQ VAS	WOMAC, VAS	WOMAC, Lequesne, OMERACT-OARSI	IKDC, KOOS, EQ VAS, Tegner score	WOMAC, SF-36	VAS, KOOS, EuroQol
Prior surgeries	No	63 Subjects	None in last year	Not recorded	101 Subjects	Not recorded	No
Prior injections	No	Not recorded	None in prior 6 months	None in prior 6 months	Conservative	None in prior 2 weeks	No
PRP no. of injections	4	3	3	3	3	2	3
PRP volume per Injection	5.5 ml	8 ml	12 ml	8 ml	5 ml	4–6 ml	5 ml
Injection interval, wk	1	1	1	2	1	4	2
Injection approach	Superolateral	Not recorded	Superolateral	Superolateral	Not recorded	Anteromedial or lateral midpatellar	Not recorded
Primary and secondary outcomes	WOMAC score before infiltration and at 4, 12, and 24 weeks after first injection	IKDC, EQ VAS, Tegner, and KOOS scores, range of motion and knee circumference changes were evaluated at 2, 6, and 12 months	WOMAC scores at 1, 2, and 6 months	WOMAC and Lequesne scores at 24 and 48 weeks	IKDC, KOOS, EQ VAS, and Tegner scores at baseline and then at 2, 6, and 12 months after last injection	WOMAC and SF-36 scores at 52 weeks	VAS, KOOS, EuroQol following the third infiltration and after 3 and 6 months following final infiltration

*Abbreviations: EQ VAS* EuroQol visual analogue scale, *IKDC* International Knee Documentation Committee, *KOOS* Knee Injury and Osteoarthritis Outcome Score, *OMERACT-OARSI* Outcome Measures in Rheumatology Osteoarthritis Research Society International, *PRP* Platelet-rich plasma, *SF-36* 36-Item Short Form Health Survey, *VAS* Visual analogue scale, *WOMAC* Western Ontario and McMaster Universities Osteoarthritis Index

**Table 2:** Safety data

Study	Adverse events
Cerza et al. [22]	No adverse reactions. None were observed in our series.
Filardo et al., 2012 [11]	Only minor adverse events were detected in some patients, such as mild pain and effusion after the injections, in particular in the PRP group, where a significantly higher post-injective pain reaction was observed (*P* = 0.039). However, this reaction was self-limiting within a few days and did not compromise the overall outcome.
Sanchez et al.. [24]	Adverse events were generally mild and evenly distributed between the groups (*P* < 0.811). Most of these adverse events (96% in the PRGF-Endoret® group and 92% in the HA group) were not related to the type of treatment.
Vaquerizo et al. [25]	Sixteen adverse events, 8 in the PRGF-Endoret® group and 8 in the HA group, were reported during the study. Adverse events were generally mild and evenly distributed between the groups (*P* = 0.610). Seven of 8 adverse events in the HA group and all the events in the PRGF-Endoret® group were related to pain associated with the infiltration.
Filardo et al., 2015 [23]	Two patients reported severe pain and swelling after HA injections, while no major adverse events were noted in the PRP group. However, PRP presented overall significantly more postinjection swelling and pain.
Raeissadat et al.......... [13]	The present authors had previously performed studies to evaluate the clinical application of PRP, and recorded safety and positive findings. It was a prospective study published in 2013 on 60 patients treated with two injections of PRP (1 every 4 weeks).
Montañez-Heredia et al. [14]	Adverse events relating to infiltration were infrequent, mild and appeared immediately, and their distribution between both groups did not show significant differences. There was pain related to infiltration in nine of 27 PRP injections and in four of 26 for HA, but only one patient (in PRP group) had transitory swelling that resolved itself. No relationship between these events and the growth factor or blood cell composition of PRP was found.

*HA* Hyaluronic acid, *PRGF-Endoret®* Plasma rich in growth factors, *PRP* Platelet-rich plasma

One study used the Ahlbäck classification system of knee OA and showed that 50.0% of patients had grade I, 36.8% had grade II, and 13.2% had grade III. Six studies used the Kellgren-Lawrence classification of knee OA and showed that 8.7% had grade I, 40.7% had grade II, 37.9% had grade III, and 12.7% had grade IV. Filardo et al. [11] reported only the average Kellgren-Lawrence grades for HA and PRP groups (2.1 and 2.2, respectively), and therefore their study was not included in the grade-percentage stratification mentioned above. Six articles reported a body mass index < 32 kg/m^2^ (26.5, 28.0, 30.9, 25.7, 27.68, and 29.7 kg/m^2^), and one article did not report the body mass index (Filardo et al. [11]). The average age ranged from 55 to 67 years.

Of the of seven articles, four studies used the WOMAC for outcome scores, two used the IKDC, three used the KOOS, one used the 36-item Short Form Health Survey, one used Tegner scoring, four used the VAS, and one used the Lequesne index.

Regardless of the outcome measures, all studies consistently demonstrated the efficacy of PRP in improving function and quality of life and reducing pain among patients with knee OA. Five studies showed that PRP is superior to HA in the treatment of knee OA, and two studies (by the same authors) showed no difference between the two treatments (Table 3).

**Table 3:** WOMAC, KOOS, Tegner, Lequesne, IKDC, and SF-36 scores

Study	Pretreatment	Early (0–6 wk)	Middle (6–12 wk)	Late (12–26 wk)	Extended (26–52 wk)
Cerza et al. [22]	ACP: WOMAC 76.9 ± 9.5	ACP: WOMAC 49.6 ± 17.7 ES: 2.8	ACP: WOMAC 39.1 ± 17.8	ACP: WOMAC 36.5 ± 17.9	DNC
		HA: WOMAC 55.2 ± 12.3	ES: 4.0	ES: 4.3	
	HA: WOMAC 75.4 ± 10.7	ES: 1.9 (*P* < 0.001) between groups	HA: WOMAC 57 ± 11.7	HA: WOMAC 65.1 ± 10.6	
			ES: 1.7 (*P* < 0.001) between groups	ES: 1.0 (*P* < 0.001) between groups	
Filardo et al. [11]	PRP: IKDC score 50.2 ± 15.7	DNC	PRP: IKDC score 62.8 ± 17.6 ES: 0.8	PRP: IKDC score 64.3 ± 16.4 ES: 0.9	PRP: IKDC score 64.9 ± 16.8 ES: 0.9
	KOOS symptoms 64.0 ± 17.9		KOOS symptoms 71.9 ± 17.0 ES: 0.4	KOOS symptoms 73.0 ± 18.3 ES: 0.5	KOOS symptoms 71.3 ± 17.9 ES: 0.4
	Pain 65.4 ± 17.7		Pain 71.9 ± 17.0 ES: 0.4	Pain 74.2 ± 19.6 ES: 0.5	Pain 74.0 ± 19.4 ES: 0.5
	ADL 69.9 ± 20.0		ADL 81.2 ± 17.9 ES: 0.6	ADL 79.1 ± 19.0 ES: 0.5	ADL 77.9 ± 20.6 ES: 0.4
	Sport 37.6 ± 24.7		Sport 48.8 ± 25.9 ES: 0.5	Sport 48.7 ± 29.5 ES: 0.5	Sport 47.4 ± 28.2 ES: 0.4
	QOL 34.9 ± 18.8		QOL 48.8 ± 25.9 ES: 0.7	QOL 48.0 ± 23.1 ES: 0.7	QOL 50.5 ± 22.6 ES: 0.8
	Tegner score 2.9 ± 1.4				Tegner score 3.8 ± 1.3 ES: 0.6
	HA: IKDC score 47.4 ± 15.7		HA: IKDC score 61.4 ± 16.2	HA: IKDC score 61.0 ± 18.2	HA: IKDC score 61.7 ± 19.0
			ES: 0.9	ES: 0.9	ES: 0.9
	KOOS		KOOS	KOOS	KOOS
	Symptoms 67.8 ± 15.7		Symptoms 71.6 ± 16.3 ES: 0.2	Symptoms 74.3 ± 16.0 ES: 0.4	Symptoms 74.2 ± 17.5 ES: 0.4
	Pain 63.1 ± 17.4		Pain 71.1 ± 18.6 ES: 0.5	Pain 73.2 ± 18.1 ES: 0.6	Pain 74.0 ± 19.4 ES: 0.6
	ADL 67.8 ± 21.0		ADL 78.2 ± 17.4 ES: 0.5	ADL 77.3 ± 18.6 ES: 0.5	ADL 77.3 ± 19.8 ES: 0.5
	Sport 34.2 ± 23.9		Sport 45.0 ± 24.1 ES: 0.5	Sport 44.7 ± 27.8 ES: 0.5	Sport 46.6 + −27.9 ES: 0.5
	QOL 33.6 ± 18.0		QOL 45.5 ± 23.9 ES: 0.7	QOL 48.5 ± 24.7 ES: 0.8	QOL 49.2 ± 26.0 ES: 0.9
	Tegner score 2.6 ± 1.2				Tegner score 3.4 ± 1.6 ES: 0.7
					*P* values not recorded
Sanchez et al. [24]	PRGF: WOMAC	DNC	DNC	PRGF: WOMAC 74.0 ± 42.7 ES: 1.1	DNC
	121.8 ± 44.4			38.2% of patients had 50% decrease in WOMAC pain score 57.3% of patients had 20% decrease in WOMAC pain score	
	Lequesne 9.5 ± 3.0			Lequesne 5.2 ± 3.4 ES: 1.4	
	HA: WOMAC			HA: WOMAC 78.3 ± 48.1	
	115.6 ± 45.1			ES: 0.8	
				24.1% of patients had 50% decrease in WOMAC pain score, 52.9% of patients had 20% decrease in WOMAC pain score	DNC
	Lequesne 9.1 ± 3.2			Lequesne 5.4 ± 3.3 ES: 1.2	
				Differences between PRGF and HA for 50% decrease in WOMAC pain score (*P* = 0.044), for 20% decrease (*P* = 0.555), for total WOMAC score (*P* = 0.561), and for Lequesne score (*P* = 0.714)	
Vaquerizo et al. [25]	PRGF: WOMAC 45.9 ± 12.7 Lequesne 12.8 ± 3.8 HA: WOMAC 50.8 ± 18.4 Lequesne 13.1 ± 38	DNC	DNC	For patients with 30% decrease in: WOMAC summed score: rate of response of PRGF was 66, 43, and 23 percentage points higher than that of HA for pain, physical function and stiffness, respectively (*P* < 0.001, *P* < 0.001, *P* = 0.02, respectively). Lequesne score: PRGF group is 56 percentage points higher than HA group (*P* < 0.001) For patients with 50% decrease in: WOMAC summed score: rate of response of PRGF was 43, 29, and 19 percentage points higher than that of HA for pain, physical function and stiffness, respectively (*P* < 0.001, *P* = 0.001, *P* = 0.035, respectively). Lequesne score: PRGF group is 25 percentage points higher than HA group (*P* = 0.002)	For patients with 30% decrease in: WOMAC summed score: rate of response of PRGF was 46, 37, and 40 percentage points higher than that of HA for pain, physical function and stiffness, respectively (*P* < .001, *P* < .001, *P* < 0.001, respectively). Lequesne score: PRGF group 46 percentage points higher than HA group (*P* < 0.001) For patients with 50% decrease in: WOMAC summed score: rate of response of PRGF was 29, 31, and 28 percentage points higher than that of HA for pain, physical function and stiffness, respectively (*P* < 0.001, *P* < 0.001, *P* = 0.001, respectively). Lequesne score: 19 and 2 percentage points in the PRGF and HA groups, respectively
Filardo et al. [23]	PRP: IKDC score 52.4 ± 14.1	DNC	PRP: IKDC score 63.2 ± 16.6 ES: 0.8	PRP: IKDC score 65.0 ± 16.1 ES: 0.9	PRP: IKDC score 66.2 ± 16.7 ES: 1.0
	KOOS Symptoms 65.5 ± 16.6		KOOS Symptoms 72.9 ± 17.0 ES: 0.4	KOOS Symptoms 74.7 ± 16.9 ES: 0.6	KOOS Symptoms 73.9 ± 17.2 ES: 0.5
	Pain 66.1 ± 17.9		Pain 73.8 ± 19.9 ES: 0.4	Pain 74.7 ± 19.3 ES: 0.5	Pain 74.9 ± 19.3 ES: 0.5
	ADL 70.6 ± 19.4		ADL 79.0 ± 19.8 ES: 0.4	ADL 79.1 ± 19.6 ES: 0.4	ADL 78.4 ± 20.7 ES: 0.4
	Sport 37.9 ± 25.0		Sport 48.0 ± 26.1 ES: 0.4	Sport 49.6 ± 28.6 ES: 0.5	Sport 49.3 ± 28.6 ES: 0.5
	QOL 36.0 ± 19.4		QOL 48.4 ± 23.1 ES: 0.6	QOL 49.2 ± 23.4 ES: 0.7	QOL 50.8 ± 24.0 ES: 0.8
	EQ VAS score 73.2 ± 12.0		EQ VAS score 76.3 ± 12.7	EQ VAS score 76.2 ± 12.9	EQ VAS score 77.6 ± 11.1
			ES: 0.3	ES: 0.3	ES: 0.4
	Tegner score 2.9 ± 1.3		Tegner score3.6 ± 1.4 ES: 0.5	Tegner score 3.7 ± 1.5 ES: 0.6	Tegner score 3.7 ± 1.3 ES: 0.6
	ROM 129.6 ± 12.2		ROM 130.6 ± 11.8	ROM 130.3 ± 10.7	ROM 130.2 ± 11.1
	TPC 410.0 ± 34.3		TPC 411.4 ± 35.2	TPC 407.2 ± 35.6 ES: 0.1	TPC 402.3 ± 33.4 ES: 0.1
	HA: IKDC score 49.7 ± 13.0		HA: IKDC score 63.5 ± 15.2 ES: 0	HA: IKDC score 63.5 ± 17.1 ES: 0	HA: IKDC score 64.2 ± 18.0 ES: 0
	KOOS Symptoms65.8 ± 16.3		KOOS Symptoms 70.9 ± 16.6 ES: 0.3	KOOS Symptoms 72.7 ± 17.4 ES: 0.4	KOOS Symptoms73.9 ± 18.4 ES: 0.5
	Pain 64.1 ± 16.5		Pain 72.6 ± 17.9 ES: 0.5	Pain74.8 ± 17.6 ES: 0.7	Pain 75.4 ± 19.0 ES: 0.7
	ADL 68.2 ± 20.2		ADL 78.0 ± 17.9 ES: 0.5	ADL78.4 ± 18.6 ES: 0.5	ADL 78.4 ± 19.3 ES: 0.5
	Sport 35.7 ± 24.6		Sport 44.0 ± 25.5 ES: 0.3	Sport 45.1 ± 27.0 ES: 0.4	Sport 46.3 ± 28.1 ES: 0.4
	QOL 35.7 ± 18.2		QOL 47.7 ± 22.1 ES: 0.7	QOL 49.9 ± 23.1 ES: 0.8	QOL 50.9 ± 24.4 ES: 0.8
	EQ VAS score 71.6 ± 13.4		EQ VAS score 73.9 ± 13.7 ES: 0.2	EQ VAS score74.1 ± 15.1 ES: 0.2	EQ VAS score 73.4 ± 15.2 ES: 0.1
	Tegner score 2.8 ± 1.3		Tegner score3.3 ± 1.5 ES: 0.4	Tegner score 3.5 ± 1.5 ES: 0.5	Tegner score 3.4 ± 1.5 ES: 0.5
	ROM 128.2 ± 12.2		ROM 129.0 ± 10.9	ROM 128.0 ± 11.4	ROM 127.4 ± 12.0
	TPC 415.0 ± 34.7		TPC 413.3 ± 34.1	TPC 408.7 ± 32.5	No statistical significance between groups
	No statistical significance between groups		No statistical significance between groups	No statistical significance between groups	
Raeissadat et al. [13]	PRP: WOMAC 39.5 ± 17.06	DNC	DNC	DNC	PRP: WOMAC 18.44 ± 14.35 (*P* < 0.001) ES: 1.2
	Pain 8.46 ± 4.17				Pain 4.03 ± 3.36 (*P* < 0.001) ES: 1.1
	Physical function 2.2 ± 1.76				Physical function 1.19 ± 1.4 (*P* < 0.001) ES: 0.6
	Stiffness 28.91 ± 12.63				Stiffness 13.19 ± 10.39 (*P* < 0.001) ES: 1.2
	SF-36 (PCS) 178.14 ± 81.0				SF-36 (PCS) 255.96 ± 77.59 (*P* < 0.001) ES: 1.0
	SF-36 (MCS) 229.22 ± 95.62				SF-36 (MCS) 269.92 ± 91.48 (*P* < 0.001) ES: 0.4
	HA: WOMAC 28.69 ± 16.69 pain 6.91 ± 3.82 physical function 1.88 ± 1.72 stiffness 19.88 ± 12.32 SF-36 (PCS) 180.4 ± 68.52 SF-36 (MCS) 226.43 ± 97.39				HA: WOMAC 27.46 ± 16.36 (*P* = 0.78) pain 5.08 ± 3.71 (*P* = 0.029) ES: 0.5 physical function 2.14 ± 1.66 (*P* = 0.16) stiffness 19.51 ± 11.9 (*P* = 0.919) SF-36 (PCS) 189.39 ± 103.73 (*P* = 0.37) SF-36 (MCS) 216.91 ± 100.9 (*P* = 0.74) ES: 0.1
Montañez-Heredia et al. [14]	DNC	PRP: EQ Worsening 7.4%	DNC	PRP: EQ Worsening 3.7%	PRP: EQ Worsening 7.4%
		Similar 74.1%		Similar 48.1%	Similar 48.1%
		Improvement 18.5%		Improvement 48.1%	Improvement 44.4%
		50% decrease VAS: 55.5%		50% decrease VAS: 55.5%	50% decrease VAS: 44.4%
		HA: EQ Worsening 0%		HA: EQ Worsening 11.5%	HA: EQ Worsening 15.4%
		Similar 65.4%		Similar 53.8%	Similar 50.0%
		Improvement 34.6%		Improvement 34.6%	Improvement 34.6%
		50% decrease VAS: 57.7%		50% decrease VAS: 30.7%	50% decrease VAS: 42.3%
				KOOS: For patients with arthritis grade II, ADL at 3-month follow-up improved significantly on the KOOS scale in the PRP group as compared with the HA group (*P* = 0.040)	KOOS: At 6 months follow-up, pain decreased for arthritis grade II patients injected with PRP (*P* = 0.012) with improvements in function in daily living (*P* = 0.013) and function in sport and recreation (*P* = 0.021)

*Abbreviations: ACP* Autologous conditioned plasma, *DNC* study did not collect data during this time period, *ADL* Activities of daily living, *EQ VAS* EuroQol visual analogue scale, *ES* Effect size, *HA* Hyaluronic acid, *IKDC* International Knee Documentation Committee, *KOOS* Knee Injury and Osteoarthritis Outcome Score, *MCS* Mental Component Summary, *OMERACT-OARSI* Outcome Measures in Rheumatology Osteoarthritis Research Society International, *PCS* Physical Component Summary *PRP* Platelet-rich plasma, *QOL* Quality of life, *ROM* Range of motion, *SF-36* 36-Item Short Form Health Survey, *TPC* Transpatellar circumference, *VAS* Visual analogue scale, *WOMAC* Western Ontario and McMaster Universities Osteoarthritis Index

In one study, the two groups that reached the minimum clinically important difference also showed a statistically significant difference in WOMAC scores, with a greater effect in the PRP group [22]. Two studies reported that both groups had clinical improvement at follow-up evaluation, but the comparison between the two groups did not show a statistically significant difference in all scores evaluated [11, 23]. In the study by Sanchez et al. [24], the rate of response to PRGF-Endoret® (BTI Biotechnology Institute, Blue Bell, PA, USA) was 14.1% higher than that of HA (95% CI, 0.5–27.6; *P* = 0.044). Regarding the secondary outcome measures, the rate of response to PRGF-Endoret® was higher than that to HA in all cases, although the difference did not reach statistical significance [24].

One study showed that at 24 and 48 weeks, the rate of response to PRGF-Endoret® was significantly higher than that to HA for all parameters, including pain, stiffness, and physical function, on the WOMAC, Lequesne index, and OMERACT-OARSI scales [25]. At the 12-month follow-up, Raeissadat et al. [13] reported that the WOMAC pain score significantly improved in both the PRP and HA groups. Although all achieved the minimum clinically important difference, but the results were significantly better in the PRP group (ES, 1.1) than in the HA (ES, 0.5) group (*P <* 0.001) [13]. Montañez-Heredia et al. [14] reported that at 3 and 6 months after treatment completion, the results in the PRP group was superior to those in the HA group in terms of VAS and KOOS scores [14]. Some studies showed that PRP was not beneficial to all participants and was associated with degree of knee OA [11, 14, 22, 24].

### Risk of bias

The risk of bias in the two RCTs that contributed to the cessation meta-analysis was low across all domains [11, 24]. In the 2012 study by Filardo et al. [11], there were three uncertain risk biases. Categorization of the included studies by the nature of their design showed that all studies were at high risk of selection bias. Three of these studies did not blind participants or personnel; considering the nature of the studies, follow-up measures, and contact with researchers, these studies were found to have a risk of selection or performance bias or both. In the other studies, the lack of intervention or contact with researchers was assumed to reflect an unlikely significant performance or detection bias.

**Fig. 2:**
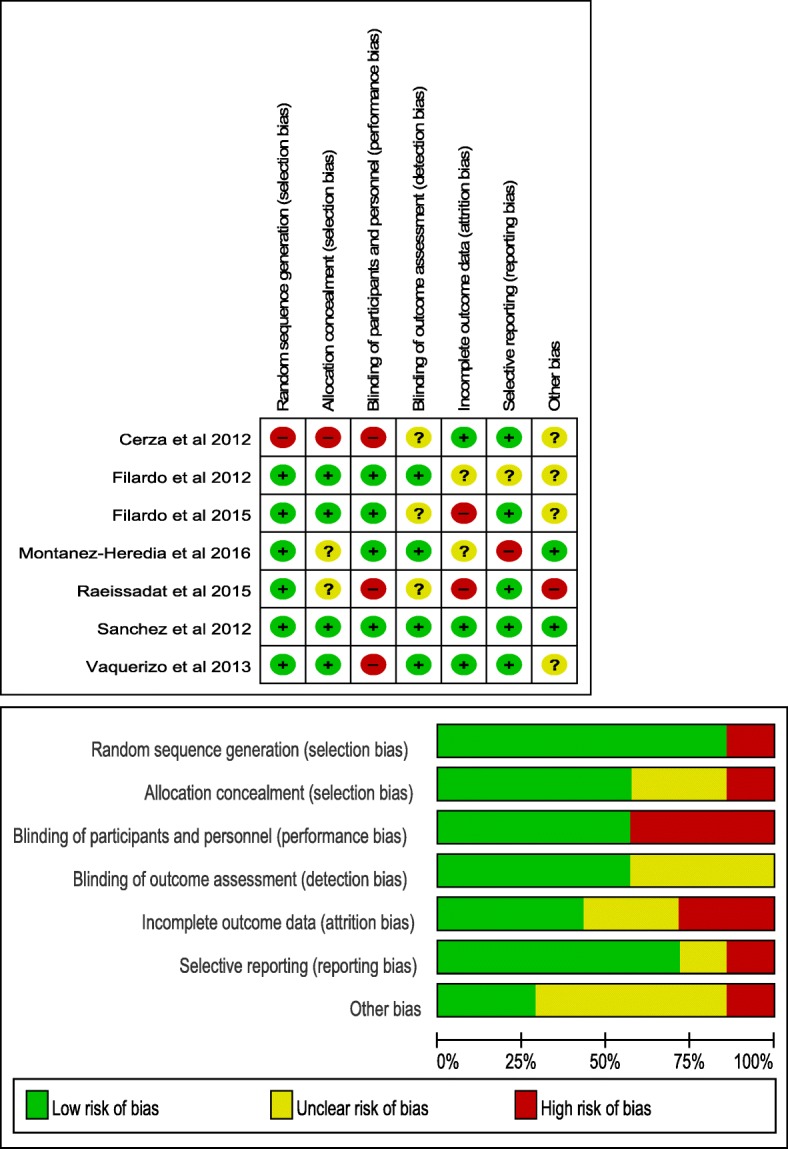
Risk-of-bias summary: review authors’ judgments about each risk-of-bias item for each included study

With regard to random sequence generation (selection bias), 85.71% of RCTs had low bias and 14.29% had high bias. With regard to allocation concealment (selection bias), 57.14% of RCTs had low bias, 28.57% had uncertain bias, and 14.29% had high bias. For blinding of participants and personnel (performance bias), 57.14% of RCTs had low bias and 42.86% had high bias. For blinding of outcome assessment (detection bias), 57.14% of RCTs had low bias and 42.86% had uncertain bias. For incomplete outcome data (attrition bias), 42.86% of RCTs had low bias, 28.57% had uncertain bias, and 28.57% had high bias. With regard to selective reporting (reporting bias), 71.43% of RCTs had low bias, 14.29% had uncertain bias, and 14.29% had high bias. Finally, for other biases, 28.57% of RCTs had low bias, 57.14% had uncertain bias, and 14.29% had high bias. Figure 2 illustrates the bias for each included study.

## Discussion

The main aim of the present study was to investigate a novel biological approach to the treatment of knee OA. In recent years, there has been an increase in the prevalence of the use of autologous blood products that might supply cellular and humoral mediators (blood growth factors) for tissue healing in a variety of applications [26]. PRP is a blood product that provides a simple, low-cost, minimally invasive alternative to obtain a concentration of many of these growth factors [27].

This systematic review shows that intra-articular injection of PRP has a modest effect in the treatment of knee OA and is superior to HA [13, 14, 22, 24, 25]. All studies except two by the same authors [11, 23] found PRP to be especially effective in patients with mild knee OA. The main findings of this systematic review are that multiple sequential intra-articular PRP knee injections (range, two to four injections) improved functional outcome scores (WOMAC) at a minimum of 24 weeks [13, 22, 24, 25]. However, no benefit of PRP was observed over the control treatment in terms of other pain measures such as IKDC, KOOS, and VAS.

With regard to the injection protocol in all studies, the present review evaluated the efficacy of once-weekly intra-articular PRP injection administered at least three times at 2–3 months after the first injection, because this regimen and time frame of PRP provide the greatest efficacy. Of the included studies, four used frozen PRP and three used fresh PRP, and four used leukocyte-poor PRP and three used leukocyte-rich PRP (Table 4). Such differences could have resulted from the preparation techniques (frequency/speed/length of centrifugation or the use of ancillary activating/anticoagulant agents), administration techniques (volume/frequency/delivery in terms of means of administration), postadministration rehabilitation protocols, participants’ baseline characteristics (age, sex, activity level, or OA grade), and the methodological rigor of the study. Safety is an important aspect of evaluating PRP as a conservative treatment. In this review, we found no serious adverse local or systemic reactions during and after injection in both the short and long term.

**Table 4:** PRP type

Study	Leukocyte-poor/rich PRP	Fresh/frozen PRP
Cerza et al. [22]	Leukocyte-poor PRP	Frozen PRP
Filardo et al. [23]	Leukocyte-rich PRP	Fresh PRP
Sanchez et al. [24]	Leukocyte-poor PRP	Fresh PRP
Vaquerizo et al. [25]	Leukocyte-poor PRP	Frozen PRP
Filardo et al. [23]	Leukocyte-rich PRP	Frozen PRP
Raeissadat et al. [13]	Leukocyte-rich PRP	Fresh PRP
Montañez-Heredia et al. [14]	Leukocyte-poor PRP	Frozen PRP

*PRP* Platelet-rich plasma

### Limitations

This study has a few limitations that need to be addressed. First, only English-language RCTs with high-grade evidence were included, which increases the risk of selection bias. Second, the pooled sample size for this review was limited, with the control arm of PRP including 460 patients and the arm control of HA including 448 patients. This small sample size can limit the power to detect changes that might reach the threshold for a minimal clinically important difference in outcome measures. The third limitation of this study is the lack of a placebo group, meaning that there is no clear evidence that PRP is indeed effective in traumatic or degenerative cartilage lesions. The majority (75%) of the overall treatment effect in OA RCTs is attributable to contextual effects rather than to the specific effect of treatments [21]. However, this review only included studies of high quality that used established outcome measures.

## Conclusions

PRP intra-articular injection of the knee may be an effective alternative treatment for knee OA, especially in patients with mild knee OA. However, some studies suggested that PRP is not more effective than HA. A large, multicenter, randomized trial study is needed to further assess the efficacy of PRP treatment for patients with knee OA.

## Abbreviations

EQ VAS: EuroQol visual analogue scale
ES: Effect size
HA: Hyaluronic acid
IKDC: International Knee Documentation Committee
KOOS: Knee Injury and Osteoarthritis Outcome Score
OA: Osteoarthritis
OARSI: Osteoarthritis Research Society International
PRGF-Endoret®: Plasma rich in growth factors technology
PRP: Platelet-rich plasma
RCT: Randomized controlled trial
WOMAC: Western Ontario and McMaster Universities Osteoarthritis Index
